# Features of patient-education websites for patients with chronic kidney disease: an analysis of recommended websites

**DOI:** 10.1186/s12882-020-02128-6

**Published:** 2020-11-03

**Authors:** Rohanit Singh, Lawrence J. Appel, Gibran Kazi, Bernard G. Jaar

**Affiliations:** 1 Welch Center for Prevention, Epidemiology, and Clinical Research, The Johns Hopkins University, 2024 E. Monument St., Baltimore, MD 21287 USA; 2 University of Charleston, 1300 MacCrokle Avenue, SE, Charleston, WV 17050 USA; 3 Division of Nephrology, Department of Medicine, The Johns Hopkins University School of Medicine, Baltimore, MD USA; 4 Nephrology Center of Maryland, Baltimore, MD USA

**Keywords:** Chronic kidney disease, Website, Internet

## Abstract

**Background:**

Chronic kidney disease (CKD) requires lifelong self-management. With the rise in access to the Internet, many CKD patients and their caregivers increasingly use the internet for information on CKD self-management. A recent environmental scan by Smekal et al. identified 11 CKD-related websites that covered the greatest number of content areas. This paper aims to evaluate these 11 selected websites in order to identify those that most effectively address content areas relevant to patients with CKD.

**Methods:**

Each website was assessed for information to 6 content areas: diet, physical activity, financial information, emotional support, general CKD information, and medication adherence. A three-tiered scoring metric was used in which a 0 was given if a content area was completely unaddressed, a (+) was given for a category that was generally addressed, and a (++) was given for a category that was addressed with actionable guidance.

**Results:**

While CKD information and diet were very comprehensively covered with scores of 11 (++) and 8 (++), respectively; physical activity, emotional support and medication adherence received the fewest (++) scores (3 for physical activity and five for both emotional support and medication adherence). For each content area, recommendations are made for websites that are particularly useful. Common themes for these highlighted websites include specific instructions, multiple modalities of information, downloadable and printable resources, and contact references for personal inquiries.

**Conclusion:**

The recommended websites can help CKD patients and caregivers utilize the most applicable information for their specific self-management needs. Website improvements related to physical activity, emotional support, and financial information for persons with CKD are warranted.

## Background

Chronic Kidney Disease (CKD) often impacts emotional, physical, and financial well-being. Because of its broad and lifelong implications, management of CKD involves a multifaceted approach to patient education and engagement that promotes general CKD information and lifestyle modications (dietary change and physical activity), maintenance of emotional and financial well-being, and medication adherence.

Currently, numerous resources are available to patients and providers, such as guidelines for CKD management, videos, modules, and clinical practice tools [1]. Studies have identified mobile health as a window of opportunity to aid patients with chronic diseases [2], with access to the Internet becoming widespread. Studies have now documented that mobile health access via computers and smartphones is increasing amongst CKD patients [3]. This greater access has allowed CKD patients and their caregivers to utilize and gather information from the Internet to aid them with their CKD self-management.

While the use of the Internet provides patients with a wide array of information, many concerns arise with regard to the quality, readability, and applicability of the information. In response to these concerns, researchers have evaluated the state of CKD-related websites and the nature of the information they provide. With the rapid dissemination and turnover of information on the Internet, increased emphasis is placed on updating the literature that CKD patients can access. The most recent environmental scan of CKD-related websites was published in July of 2019 [4]. This systematic review *Smekal et. al, 2019* assessed English CKD-related websites with respect to the number of content areas, and highlights 11 websites that address the greatest percentage of content areas [4]. While this research is useful in underscoring specific websites that cover the greatest amount of information, it does not offer patients and providers specific recommendations about which websites are best suited for delivering actionable guidance.

This paper evaluates the 11 websites from *Smekal et. al, 2019* to identify those with the most useful information in six domains relevant to CKD self-management: CKD information, dietary needs, physical activity, emotional support, financial information, and medication adherence [4]. The main objective of this paper is to provide CKD patients and healthcare providers with recommendations for websites that provide actionable guidance for CKD management.

## Methods

The 11 websites from *(Smekal et. al, 2019)* assessed in this paper are limited to English language websites and included *DaVita, National Kidney Foundation, Fresenius Kidney Care, Kidney Foundation of Canada, Kidney Care UK, Manitoba Renal Program, National Institute of Diabetes and Digestive and Kidney Diseases, Renal Support Network, The Renal Association, Life Options, NHS Inform* [4]. Each website was assessed for the extent to which it provides practical information related to CKD information, dietary needs, physical activity, emotional support, financial information, and medication adherence. The investigators of this paper selected these content areas based on their known association with CKD. The distribution modality was also noted (i.e., via text, videos, or pictures,).

For each content area, each website was assigned one score of a 0, a (+), or (++). A score of ‘0’ was given for websites that did not address a category, while a score of ‘+’ indicated that the category was addressed in a general sense but without practical guidance for CKD patients. An example of the latter score include statements to limit protein, sodium, and potassium in a CKD diet without providing guidance on acceptable foods and recipes, and approaches to eating while away from home. A score of ‘++’ was given if the website offered practical guidance relevant to that content area. Instructions and specific recommendations are examples of guidance that warranted a score of ‘++’.

Two reviewers (R.S. and G.K.) independently assessed each of the 6 domains on the 11 websites identified by *Smekal* et al. The two reviewers then adjudicated differences and assigned the final score. Results were then tabulated.

## Results

A summary of the final assessments of the 11 websites is provided in Table 1.

**Table 1:** Summary of Scores by Category for 11 Websites identified by Smekal et al.

	Diet		Physical Activity		Emotional Support		Financial Information		CKD Information		Medication Adherence	
Website	Score	Modality	Score	Modality	Score	Modality	Score	Modality	Score	Modality	Score	Modality
DaVita	++	Text, Pictures, Video	+	Text	++	Text	++	Text	++	Text, Pictures, Videos	++	Text
National Kidney Foundation	++	Text, Pictures	+	Text	++	Text	++	Text	++	Text, Pictures, Videos	++	Text
Fresinius Kidney Care	++	Text	+	Text	++	Text	++	Text	++	Text, Video, Picture	++	Text, Picture
Kidney Foundation of Canada	++	Text, Picture	++	Text, Pictures	+	Text	++	Text	++	Text, Picture	+	Text
Kidney Care UK	++	Text, Videos, Pictures	+	Text	++	Text	+	Text	++	Text, Videos, Pictures	+	Text
Manitoba Renal Program	++	Text, Videos, Pictures	+	Text	0	N/A	0	N/A	++	Text, Videos	++	Text
National Institute of Diabetes and Digestive and Kidney Diseases	++	Text, Pictures	++	Text, Pictures	+	Text	++	Text	++	Text, Videos	+	Text
Renal Support Network	++	Text, Picture	+	Text	+	Text	++	Text, Videos	++	Text, Pictures	++	Text
The Renal Association	+	Text	+	Text	+	0		N/A	++	Text	+	Text
Life Options	+	Text	++	Text, Pictures	++	0		N/A	++	Text, Videos, Pictures	+	Text
NHS Inform	+	Text	+	Text	+	+		Text	++	Text	+	Text

Notably, all 11 websites received a (++) score in the category of CKD information. All websites contained detailed information regarding symptoms of CKD, key assays and relevant laboratory values, and guidelines for monitoring CKD progression, both at home and with a provider. Eight of the 11 websites received a (++) for dietary information, with no website receiving (0). Most of the websites were highly effective in not only outlining the need for restricted intake of protein, sodium, potassium, and phosphorous, but also in providing some combination of instructional videos, recipes, dietary tracking tips and worksheets, and lists of options in grocery stores and restaurants. The high distribution of (++) scores within the CKD information and diet categories demonstrates that these domains of CKD management are comprehensively covered among the 11 websites.

The 11 websites received the fewest (++) scores in the domains of physical activity, emotional support, and medication adherence. Only three websites received a (++) score in the domain of physical activity; these sites provided instructions for specific exercises that were appropriate for CKD patients of different ages and physical abilities. While no website received a ‘0’ in this category, most websites simply pointed out that physical activity is important for CKD management without providing specific instructions, stretches, or activities for CKD patients. The categories of emotional support and medication adherence both had 5 (++) scores. While no website received a (0) for medication adherence, some programs received a 0 in the category of emotional support for failing to address that specific need.

**Fig. 1:**
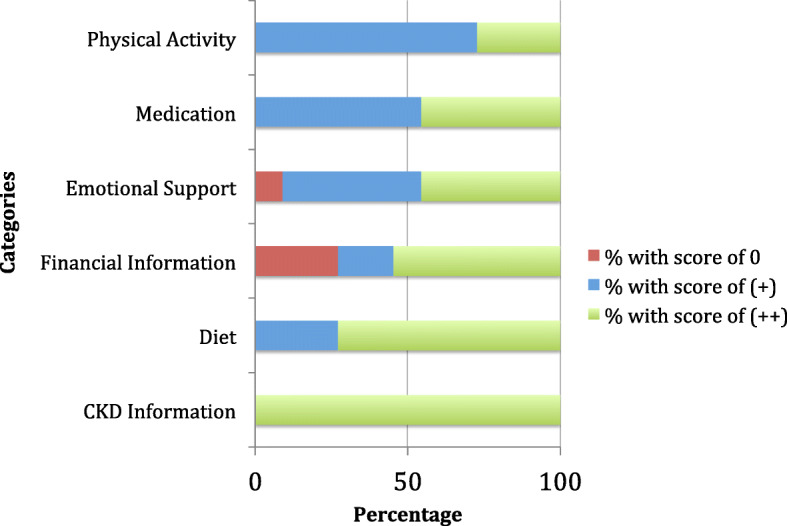
Distribution of scores within each category; Red indicates the percentage of websites with a score of 0 for specified domain; Blue indicates the percentage of websites with a score of (+) for specified domain; Green indicates the percentage of websites with a score of (++) for specified domain

The financial information category had the greatest variation in score distribution, with three websites receiving a 0, two receiving a (+), and six receiving a (++). This indicates that while financial information was left unaddressed more than any other category, it is still addressed effectively in over half of the websites. The full distribution of scores within all categories is displayed in Fig. 1.

As was noted before, physical activity and financial information were the only categories that were unaddressed by one and three websites, respectively. Physical activity had the fewest number of (++) scores. The wide range of (++) scores within diet and CKD information highlights that these categories are covered relatively more effectively than the categories of physical activity, emotional support, medication adherence, and financial information.

With regard to the modality of information provided, the category of CKD information had the greatest use of different modalities, with multiple websites utilizing a mixture of text, videos, and pictures. The category of dietary information also contained a greater use of different modalities compared to other categories, with many websites including videos, pictures, and text to provide instructions and information. Emotional support information was distributed exclusively through text. Financial information and medication adherence were also almost exclusively discussed via text, with only one website utilizing video and pictures for each category. Notably, the only websites that received a (++) for physical activity were those that provided information with both text and video, rather than text alone.

After all 11 websites were assessed, all websites that received a (++) were analyzed by category to identify those that are particularly useful for patients. The final results of this analysis with recommendations by category are provided in Table 2, which also highlights the rationale for these selections. None of the websites had a specific means of allowing patients to publicly evaluate the website or provide feedback on the quality and usefulness of the education material provided.

**Table 2:** Suggested CKD Websites by Category

Category	Website Name	Reason
**Diet**	National Kidney Foundation	Many detailed recipes with pictures and instructions; guidelines for tracking and logging macronutrient content
	Fresenius Kidney Care	Compendium of recipes with instructions and some videos; foods to avoid and suggestions for orders at restaurants and grocery stores
**Physical Activity**	Kidney Foundation of Canada	Detailed instructions and visual photo guidelines for specific exercises and techniques
	Life Options	Booklet compendium with multiple exercises, detailed instructions, and visual guidelines
**Emotional Support**	Fresenius Kidney Care	Contains specific portals for different kinds of emotional needs as well as tools for managing body image, counseling, and forming a social support group
	Kidney Care UK	Contains detailed information regarding prevalence of depression and depression screening; coping strategies and counseling details also provided
**Financial Information**	NIDDK	Large amounts of information about insurance options, payments, employment rights, and grants
**CKD Information**	DaVita	Huge array of educational materials, videos; self-paced classes sometimes with a live instructor
	Kidney Foundation of Canada	Multiple pamphlets and booklets; provides symptoms and syndromes of CKD by etiology
**Medication**	Manitoba Renal Program	Contains specific information for each medication class and how best to adhere

## Discussion

This paper, which analyzed the 11 patient-education websites from *(Smekal et. al, 2019*), identified specific websites that would be most useful to CKD patients in each of six relevant categories. While the categories of diet and CKD information were well covered [8 and 11 (++) scores respectively], the categories of physical activity, emotional support, and medication adherence were not [3, 5, and 5 (++) scores, respectively]. Financial information was unaddressed on more websites than any other category (3 (0) scores). These findings identify important gaps that should be addressed in future website updates.

Consistent with findings that CKD information and diet have the best coverage, these two categories also exhibit the greatest variety of media, with almost all websites including pictures and videos, as well as text. The importance of using a combination of modalities is heightened by the fact that the only websites receiving a (++) in physical activity were those using a combination of text and pictures, rather than text alone. Hence, in addition to being visually appealing, the variety of media also appear to convey enhanced content with actionable guidance.

Recommended websites by category (Table 2) were made based on which websites provided practical guidance. For diet, the *National Kidney Foundation* and *Fresenius Kidney Care* websites were selected, because they provide options for dietary tracking, recipes with step-by-step instructions, and multiple modalities of information sharing. For physical activity, the *Kidney Foundation of Canada* and *Life Options* were selected, as they were the only websites to provide instructions and illustrations for specific exercises, movements, and activities for patients of all ages and capabilities. For emotional support, *Fresenius Kidney Care* and *Kidney Care UK* were selected, because they provided well-defined coping strategies for specific emotional needs surrounding body image, depression, counseling, and social support. The *NIDDK* website is particularly effective for financial information, as it was the only one enabling patients to learn about employment rights, financial insurance options, grants, and options for payment of medication. While all websites were assessed to be high in quality for general CKD information, *DaVita* and *Kidney Foundation of Canada* were deemed outstanding in their presentation of CKD information in live modules and self-paced courses, with separate subcategories by types of CKD. For medication adherence, the *Manitoba Renal Program* is the preferred website, because it is the only one that classifies medications based on their class while also providing tips and reminders for taking prescribed medications.

Our study has limitations. First, it only critiques the top 11 websites as highlighted by Smekal et al., 2019 [4]. Although these websites were chosen because they were systematically evaluated and deemed to have addressed the greatest amount of content areas, it is possible that other websites may be particularly useful for certain specific categories without addressing a broad number of other content areas. Still, these websites are extremely popular websites affiliated with national organizations. Second, experienced clinicians and researchers, not stakeholders and patients, selected the categories for evaluation. Ideally, designing, evaluating, and updating patient-education websites should ideally include patients and other stakeholders. Third, in this paper, we focused on content, rather than technical aspects of the websites, such as reading level and ease of navigation. While reading level was previously assessed in the Smekal et al. paper, a formal evaluation of ease of navigation was deemed to be beyond the scope of our paper. However, we did assess one key feature as a surrogate for ease of navigation, which was whether the relevant, practical information for a domain could be found within a single link in the website (i.e. no extra searching or browsing was needed). Our principal finding is that almost all websites contained practical information under a single link for diet and CKD information (10 and 11 respectively) while physical activity, financial information, and emotional support had the least (2, 3, and 4 respectively). Medication adherence information was found under a single link in 7 different websites.

Our study also has strengths. To our knowledge, this study is the first of its kind to identify and recommend specific websites for different aspects of CKD self-management. As such, our findings should be useful in guiding patients to information sources that provide relevant, actionable guidance [5, 6]. Second, the study also highlighted gaps in content. While diet and general CKD information were well-covered categories, other content areas were not, specifically, physical activity, emotional support, and medication adherence.

## Conclusion

In conclusion, the widespread and growing use of the Internet provides an unparalleled opportunity for CKD patients to find information on disease self-management. This paper provides a guide to patients and their providers on recommended websites with a focus on specific content areas of direct relevance to CKD.

## Data Availability

All data generated or analyzed during this study are included in this published article and the provided figures and tables.
